# Glycylglycine promotes the solubility and antigenic utility of recombinant HCV structural proteins in a point-of-care immunoassay for detection of active viremia

**DOI:** 10.1186/s12934-024-02297-1

**Published:** 2024-01-18

**Authors:** Heba Shawky, Ashraf A. Tabll, Reem M. Elshenawy, Naiera M. Helmy, Rehab I. Moustafa, Yasser K. Elesnawy, Marwa M. Abdelghany, Yasmine S. El-Abd

**Affiliations:** 1 Therapeutic Chemistry Department, Pharmaceutical Industries and Drug Research Institute, National Research Centre, Dokki, Cairo, 12622 Egypt; 2 Microbial Biotechnology Department, Biotechnology Research Institute, National Research Centre, Dokki, Cairo, 12622 Egypt; 3 National Committee for Control of Viral Hepatitis (NCCVH), Ministry of Health and Population, Cairo, Egypt; 4 Ahmed Maher Teaching Hospital, Cairo, Egypt

**Keywords:** Glycylglycine, *E. coli* (BL21), Chaperone, HCV, Enzyme immunoassay, Core, E1/E2, Viremia

## Abstract

**Background:**

Although *E. coli* is generally a well-opted platform for the overproduction of recombinant antigens as heterologous proteins, the optimization of expression conditions to maximize the yield of functional proteins remains empirical. Herein, we developed an optimized *E. coli* (BL21)-based system for the overproduction of soluble immunoreactive HCV core/envelope proteins that were utilized to establish a novel immunoassay for discrimination of active HCV infection.

**Methods:**

The core/E1-E2 genes were amplified and expressed in *E. coli* BL21 (DE3) in the absence/presence of glycylglycine. The antigenic performance of soluble proteins was assessed against 63 HCV-seronegative (Ab^−^) sera that included normal and interferent sera (HBV and/or chronic renal failure), and 383 HCV-seropositive (Ab^+^) samples that included viremic (chronic/relapsers) and recovered patients’ sera. The color intensity (OD4_50_) and S/Co values were estimated.

**Results:**

The integration of 0.1–0.4M glycylglycine in the growth media significantly enhanced the solubility/yield of recombinant core and envelope proteins by ~ 225 and 242 fold, respectively. This was reflected in their immunoreactivity and antigenic performance in the developed immunoassay, where the soluble core/E1/E2 antigen mixture showed 100% accuracy in identifying HCV viremic sera with a viral RNA load as low as 3800 IU/mL, without cross-reactivity against normal/interferent HCV-Ab^−^sera. The ideal S/Co threshold predicting active viremia (> 2.75) showed an AUC value of 0.9362 (95% CI: 0.9132 to 0.9593), with 87.64, 91.23% sensitivity and specificity, and 94.14, 82.11% positive and negative predictive values, respectively. The different panels of samples assayed with our EIA showed a good concordance with the viral loads and also significant correlations with the golden standards of HCV diagnosis in viremic patients. The performance of the EIA was not affected by the immunocompromised conditions or HBV co-infection.

**Conclusion:**

The applicability of the proposed platform would extend beyond the reported approach, where glycylglycine, low inducer concentration and post-induction temperature, combined with the moderately-strong constitutive promoter enables the stable production of soluble/active proteins, even those with reported toxicity. Also, the newly developed immunoassay provides a cost-effective point-of-care diagnostic tool for active HCV viremia that could be useful in resource-limited settings.

## Background

Egypt has long endured the enormous health, social and economic impacts of hepatitis C infection, which motivated the launching of a national treatment programme “*100 million seha*” in 2014 in line with global targets, aiming at reducing the prevalence to < 2% within one decade [1]. With the advent of highly efficacious oral direct-acting antivirals (DAAs), Egypt has witnessed a significant increase in the cure rates that exceeded 90%, especially in combination with ribavirin [2]. Still, the large number of currently ongoing infections worldwide estimated at 1.5 million new infections per year [3] besides the unavailability of prophylactic vaccine mandate prioritizing the preventive measures, particularly screening, at the same level as the treatment campaigns. Early diagnosis of HCV infection is not only advantageous for minimizing the risk of liver-related morbidity/mortality; it also serves to reduce the pool of infected persons and thereby restricts further transmission [4]. In general, HCV infection is primarily diagnosed by serological detection of anti-HCV antibodies (IgG) in serum samples, either by enzyme immunoassays (EIA) or chemiluminescence immunoassays (CIA) [5]. Although both assays have the same immunological basis for detection, EIAs are reportedly more sensitive and specific than CIAs in the context of blood transfusion screening [6, 7]. However, the performance of both types can be affected by regional HCV genotypes and nucleotide/amino acid polymorphisms, which leaves a considerable number of suspected HCV infections “misdiagnosed” showing false negative results. This drawback is particularly pronounced in cases with low viremia and/or during the window period of acute infection, or immunocompromised patients including those with major immunosuppression conditions like advanced HIV infection or organ transplant recipients, and chronic renal failure (CRF)-patients on long-term hemodialysis [8, 9]. In another vein, the 4th generation anti-HCV serologic tests lack the ability to discriminate viremic HCV (V-HCV) from resolved (R-HCV) infection, which might lead to another aspect of misdiagnosis; *i.e*. false positive results [10, 11]. On the other side, the applicability of the RT-PCR golden standard for viremic HCV diagnosis is limited by the high cost and expertise/infrastructure prerequisite, particularly in developing regions [12].

Against this background, seeking efficient and cost-effective HCV diagnostics have been actively pursued in myriad studies that highlighted the clinical utility of recombinant antigens, either as full-length or pre-selected mono-/multiple epitope(s)-based peptides [13–15]. In this domain, *Escherichia coli*-based system is the most widely used for the overexpression of recombinant antigens, given its high growth rate, relatively cheap growth media, and the high yield of recombinant proteins synthesized [16]. However, the bacterial expression of heterologous proteins is often challenging, as in most cases they are expressed as insoluble aggregates of misfolded proteins, *i.e*. inclusion bodies, which significantly impacts their anticipated biological activity [17]. While the recovery of soluble proteins from inclusion bodies is usually laborious and affects the ultimate yield and specific activity [18], several strategies have been approached to circumvent the misfolding and aggregation of overexpressed proteins in *the E. coli* system, including decelerating the expression rate by reducing the inducer concentration and induction temperature, or using weak promoter-based expression vectors, or targeting the improvement of the solubility of expressed proteins per se, through the co-expression of molecular chaperones [19]. Notwithstanding, all of these approaches failed to achieve the balance between the high yield of soluble protein with maintained specific activity, where suboptimal growth temperatures affected the ultimate protein yield, and the recombinant protein was still expressed as insoluble aggregates [20, 21], while the uncontrolled co-expression of molecular chaperones was associated with undesirable effects on the protein yield/stability [22]. Another approach that showed some success in maintaining the quality control of the soluble protein expression process included the integration of compounds with chemical chaperones-like behavior in the growth cultures. Among those compounds, glycylglycine dimer reportedly enhanced the solubility and yield of recombinant proteins with no effect on their biological activity [23, 24]. In this study, we developed an optimized platform for the overproduction of soluble HCV structural proteins (core and envelope (E1/E2)) in *E. coli* BL21, and assessed their antigenic utility in a point-of-care immunoassay for accurate diagnosis of active HCV viremia.

## Results

### Amplification of target genes and construction of the expression system

**Fig. 1:**
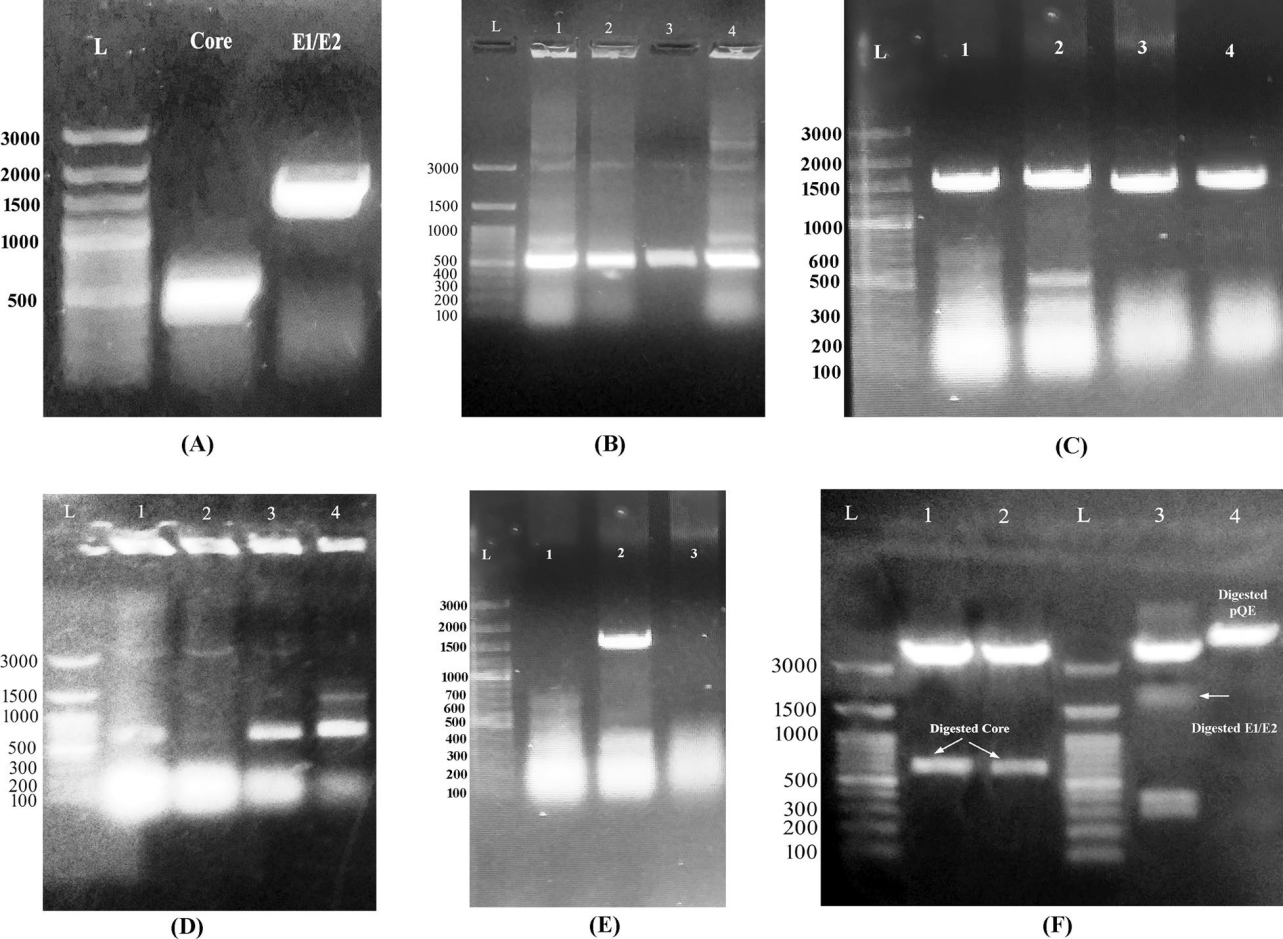
Amplification of HCV structural genes. The core and E1/E2 fragments were visualized at the expected M.wt of ~ 573 and 1665 bp, respectively (**A**). All the screened recombinant colonies showed the target genes at the expected M.wt (**B**, **C**). Three core clones were in the proper orientation for subsequent digestion (**D**), while only one E1/E2 clone was properly oriented (**E**). The cloned fragments were released from the PCR vector, and similarly, the expression vector (pQE-Trisystem) was linearized using FastDigest *KpnI* and *NotI* restriction enzymes for subsequent ligation and protein expression (**F**)

HCV-core and E1/E2 gene fragments were successfully amplified (Fig. 1A) and cloned in the PCR cloning vector (Fig. 1B, C) as indicated by agarose gel electrophoresis of the amplicons and the recombinant clone screening. Both gene fragments were visualized at the expected length, ~ 573 bp (nucleotide position: 341–913) and 1665 bp (nucleotide position: 914–2576), respectively. For the construction of the expression system, the recombinant colonies bearing both genes were first screened for proper orientation using M13 and specific gene primers. Three of the four core transformants were in the proper orientation, while one E1/E2 clone was in the correct orientation (Fig. 1D, E). The core and E1/E2 fragments were released by restriction digestion (Fig. 1F) and subsequently cloned in the digested pQE-Tris system for protein expression. Notably, a limited number of recombinant E1/E2 transformants was observed compared to those of core, which interprets the scarcity of correctly oriented E1/E2 clones.

### Analysis of the nucleotide sequence of the amplified genes

**Fig. 2:**
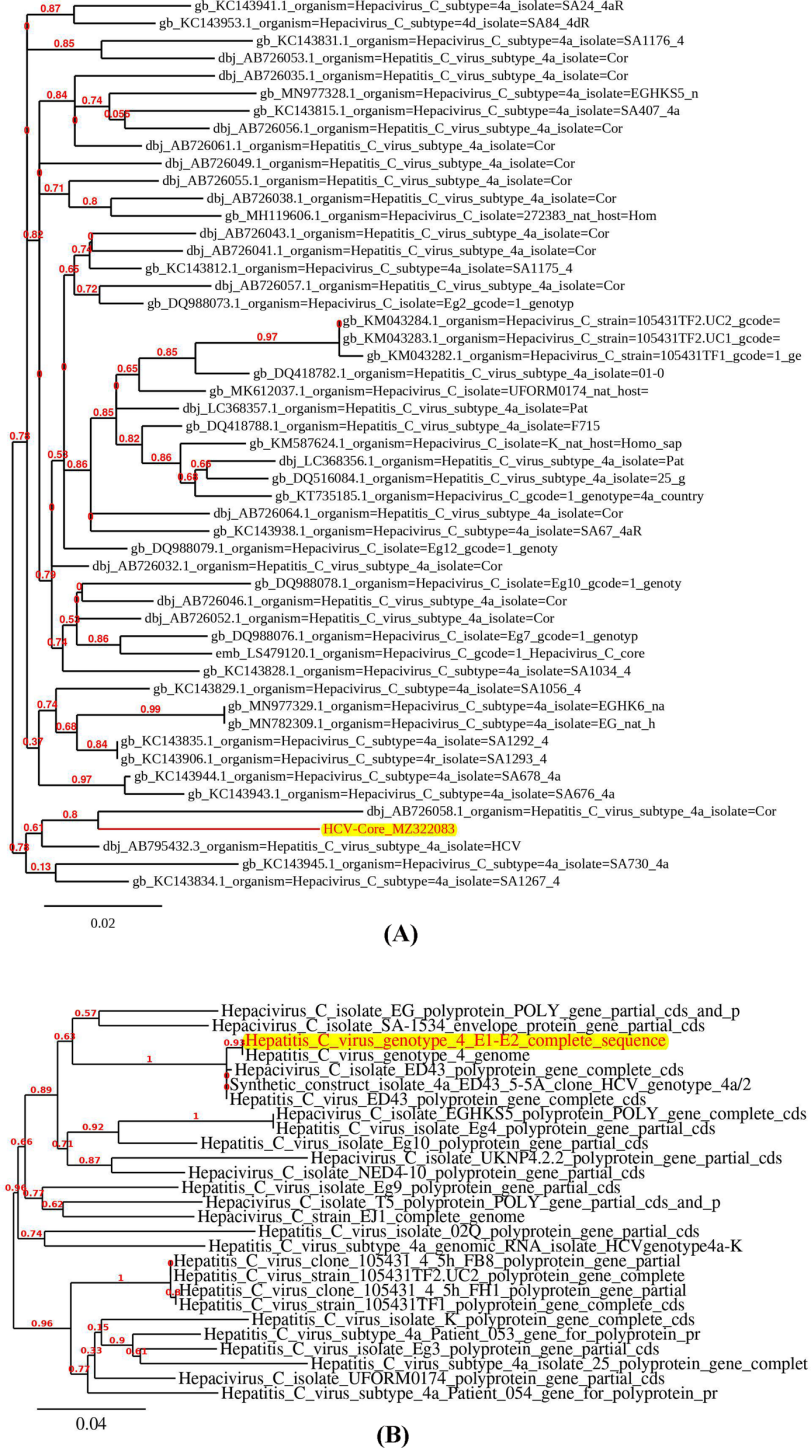
Phylogenetic comparison of amplified HCV core (**A**) and E1/E2 (**B**) nucleotide sequences with published sequences with the highest degree of homology

As demonstrated in the phylogenetic tree **(**Fig. 2**)**, the BLAST results of the core and E1/E2 gene sequences showed a partial homology with the previously published sequences, with the highest nucleotide/amino acid sequence homology of 95.45, 99%, and 89.01, 98%, respectively. The consensus sequences of the core and E1/E2 fragments were submitted to the NCBI database under the accession numbers OQ029379.1 and OQ029380.1, respectively.

### Optimization of *protein expression and characterization of* soluble recombinant proteins

**Fig. 3:**
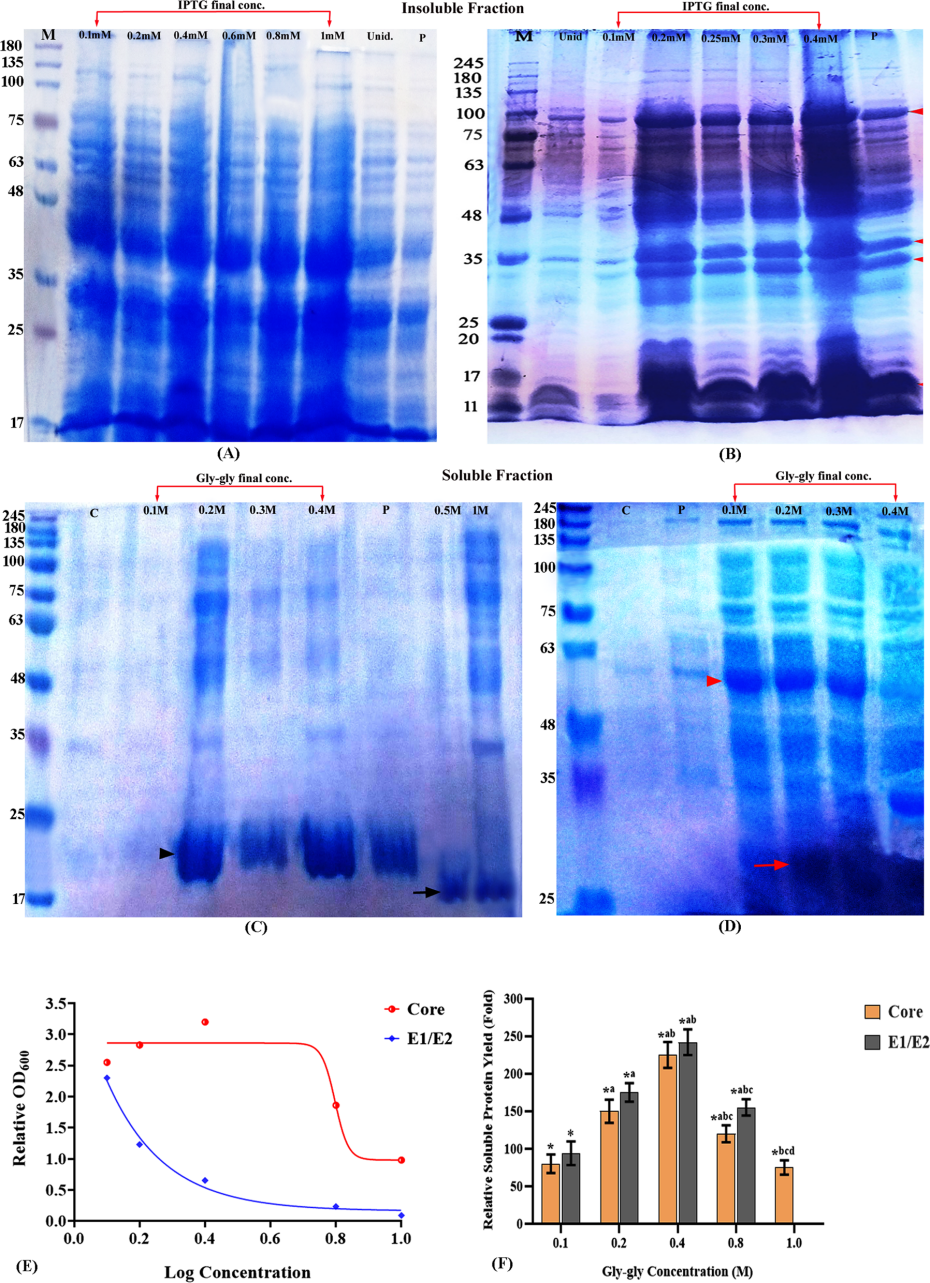
Biochemical characterization of denatured and soluble recombinant proteins. The cell lysates of recombinant cultures induced with different IPTG concentrations were resolved on 16 and 14% SDS‒PAGE to assess the expression of HCV-core (**A**, **C**) and E1/E2 (**B**, **D**), respectively. Multiple protein bands were visualized in both insoluble protein profiles, where prominent bands of core protein were detected at molecular weights range ~ 30–45 kDa, whereas E1/E2 was visualized as a dominant band at ~ 90kDa with minor bands at lower M. wts (red arrow heads). The inclusion of glycylglycine improved the solubility of recombinant core and E1/E2, where both were visualized at the expected M. wt. of ~ 21 (black arrowheads) and 61 kDa (red arrowheads), respectively compared with the glycylglycine-free cultures (lane C). The protein profiles showed that 0.4 and 0.1M final concentration of glycylglycine produced the highest yield of soluble core and E1/E2, respectively, while higher concentrations produced a lower M.wt. protein forms (black and red arrows, respectively**)**, and therefore they were used for protein purification (lane P).The integration of glycylglycine in the culture media also enhanced the cell density (**E**) and purified protein yield (**F**) in a concentration-dependent manner

Serial dilutions of IPTG inducer (0.1-1mM final concentration) were assessed to determine the lowest final concentration that induces the highest soluble recombinant proteins. Induced cultures were resolved by SDS‒PAGE to assess the protein profile, and the results showed that 1 mM IPTG was the most appropriate for the expression of HCV-core **(**Fig. 3**A),** while 0.2–0.4 mM IPTG suited the toxic nature of the E1/E2 protein **(**Fig. 3B). Despite the optimization of expression conditions in glycylglycine-free core and E1/E2 cultures, which included several IPTG concentrations and low pre/post-induction incubation temperatures (30 and 16℃, respectively), the protein profiles visualized on the SDS‒PAGE showed several bands of both target proteins at higher M.wt than expected, where predominant core bands were detected at M.wt range ~ 30-45kDa (Fig. 3A), while the recombinant E1/E2 protein was visualized as a dominant band at ~ 90 kDa with multiple bands at lower M. wt. (3B), suggesting that the rate of expression still favors the accumulation of recombinant proteins in insoluble inclusion bodies. Meanwhile, the glycylglycine-solubilized core (3C) and E1/E2 (3D) proteins were visualized as prominent bands at the expected M.wts of ~ 21 and 61kDa, respectively. However, other forms of the target proteins were observed at lower M. wts as the dimer concentration increased. In detail, the 0.4 and 0.1M glycylglycine-supplemented core and E1/E2 cultures, respectively, displayed the best production of the soluble protein with the minimal background. However, the core cultures supplemented with higher glycylglycine concentrations showed the major band at a lower M. wt. (~ 17kDa), and similarly, the SDS-PAGE profile of E1/E2 cultures containing > 0.2M concentrations showed lower M.wt. forms of the target protein within the range of ~ 26-45kDa. The integration of glycylglycine significantly also promoted the growth of recombinant cultures, particularly those expressing E1/E2 protein, as indicated by the higher cell density compared with the core and glycylglycine-free cultures **(**3E). In the same line, it was observed that the soluble protein yield was proportional to the dimer concentration within the range of 0.1–0.4M, with the highest yield (~ 225 and 242 fold for core and E1/E2, respectively) obtained in those containing 0.4 and 0.1M concentration (*P* < 0.0001), then a sharp decline of protein content was observed in cultures with dimer concentration > 0.4M (3F).

### Assessment of specific activity of soluble recombinant proteins

#### Immunoreactivity & antigenicity

**Fig. 4:**
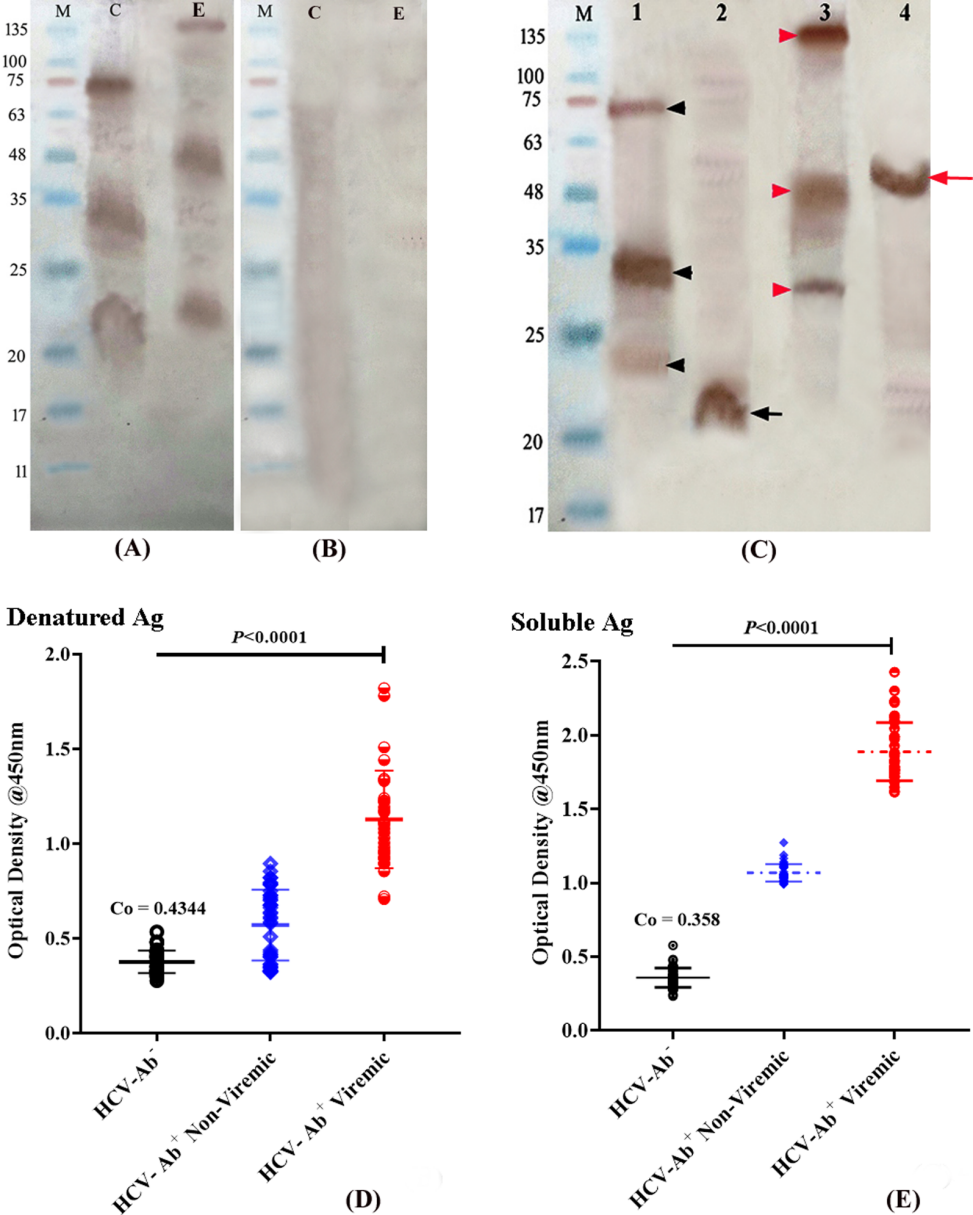
Immunoreactivity of denatured and soluble recombinant proteins. The crude preparations of denatured core (4**A**: lane **C**, **4**C: lane 1) showed immunogenic bands at M. wt. of 24, 33 and 74 kDa, while E1/E2 protein bands **(**4**A**: lane E, **4**C: lane 3) were visualized at M.wt. of 135 kDa, in addition to lower M. wt. bands at ~ 50 and 33kDa, whereas no immunogenic bands were visualized when both protein preparations (crude) were tested against control seronegative serum (4**B**). The purified preparations of soluble core and E1/E2 proteins demonstrated prominent immunogenic bands at the expected molecular weight of ~ 21 and 90 kDa, (4**C**: lane 2 and 4) respectively (black and red arrows). Lane (M) refers to the standard protein marker (11–245 kDa). Figure (4**D**) and (4**E**): Scattered plots showing the immunoreactivity of the denatured and soluble coating antigen mixtures to the corresponding antibodies in patients’ sera. All of the viremic/ non-viremic sera (*n* = 42/each) assayed against the denatured and soluble antigens showed OD_450_ values > the cutoff value of 0.4344 and 0.358, respectively, while the seronegative sera showed values ≤ cutoff value

The purified preparations of insoluble and soluble protein were assessed for immunoreactivity using HCV-patients’ sera as a source of detector antibodies in western blot. As demonstrated in Fig. 4**A**–**C)**, the immunogenic bands of denatured core and E1/E2 recombinant proteins were visualized at different molecular weights. The Ab^+^—spiked core protein showed two prominent bands at M. wt. of ~ 33 and 74 kDa in the crude preparation besides a moderately dense band that appeared around the expected size at ~ 24 kDa, while the E1/E2 bands were prominently visualized at M.wt. of 135 kDa, in addition to lower M. wt. bands at ~ 50 and 33kDa. Meanwhile, no immunoreactive bands were visualized in the crude protein preparations when tested against control HCV-seronegative serum (4B). In contrast, the immunogenic bands of soluble antigens were visualized as single homogenous bands corresponding to the expected M. wt. with minimal background in line with the SDS-PAGE profiles. For assessment of specific antigenicity, we established an in-house immunoassay using both the denatured and soluble recombinant proteins as coating antigens against HCV-seropositive (Ab^+^) viremic/non-viremic patients and seronegative (Ab^−^) healthy individuals (*n* = 42/each). The immunoassay was optimized and standardized, and results showed that 250 and 200 ng/well of denatured and soluble core-E1/E2 mixture (1:1 ratio), and 1:300, 1:1600 serum dilutions were most appropriate for anti-HCV antibodies detection without cross reactivity, respectively. All of the RNA ( ±) seropositive samples tested against the denatured proteins showed OD_450_ values > 0.4344, where the viremic sera showed an OD_450_ range of 0.708 – 1.82, while the non-viremic sera showed an OD_450_ range of 0.322 – 0.895 (Fig. 4B). The same samples showed OD_450_ values > 0.358 when assayed against the soluble antigens, with viremic and non-viremic sera showing ranges of 1.614 – 2.428 and 0.991 – 1.273, respectively (4C).

### The antigenic performance

**Fig. 5:**
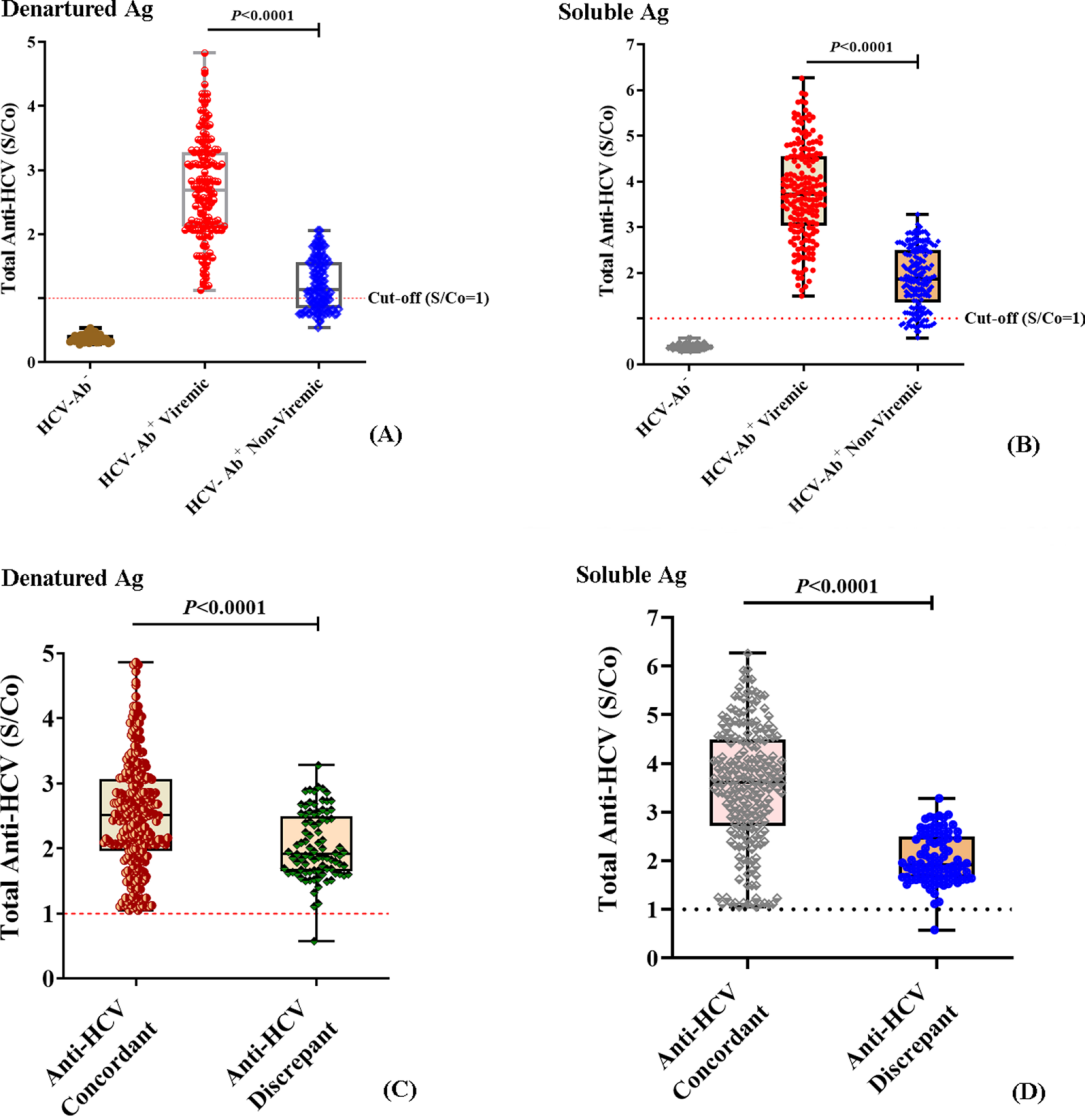
Performance evaluation of recombinant antigens in the newly developed anti-HCV immunoassay. Patients’ sera were assayed using the optimized EIA format, and S/Co values were calculated. All viremic sera (*n* = 190) besides 326 (85.12%) and 359 (93.75%) responder sera tested against both the denatured **A** and soluble antigens **B** showed OD_450_ values > S/Co threshold (2.75), while 14.88% (*n* = 57) and 6.25% (*n* = 25) of the Ab^+^-non-viremic sera of DAA-responders and/or immunocompromised patients showed OD_450_ values < S/Co threshold (2.75), respectively. **C**, **D**: The concordance of S/Co values of patients’ sera assayed against denatured and soluble antigens, respectively, where 80.04% (*n* = 357) and 89.01% (*n* = 397) of tested samples were concordant with the findings of the viral RNA quantification results, whereas 19.95% (*n* = 89) and 10.98% (*n* = 49) were anti-HCV discrepant

The OD_450_ values of all sera assayed against the denatured and soluble antigen mixtures were normalized by calculating the signal/cutoff (S/Co) values to compare the antigenic performance of denatured and soluble antigens against a large sample size. To achieve this, different sera panels were carefully selected to encompass HCV-seropositive (Ab^+^) (*n* = 383), HCV-seronegative (Ab^−^) normal individuals (*n* = 50), in addition to HCV-Ab^−^ interferent samples (HBV^+^ and/or CRF (*n* = 13)) to assess the sensitivity and specificity/cross-reactivity of both antigen preparations. The sample details of the evaluation panels are listed in Table 1. Results revealed a positive reactivity of all viremic sera (*n* = 190) besides 85.12% (*n* = 326) and 93.75% (*n* = 359) of Ab^+^-non-viremic sera (DAA-responders) tested against both the denatured (Fig. 5A) and soluble antigens (Fig. 5B) with S/Co values > threshold (2.75), respectively, while 14.88% (*n* = 57) and 6.25% (*n* = 25) of the Ab^+^-non-viremic sera, mostly belonging to DAA-responder and/or immunocompromised patients, showed S/Co values < threshold (2.75), respectively. The mean S/Co value of viremic sera tested against denatured and soluble antigens was 6.22 and 10.48-fold higher relative to normal sera, with a range of 1.125 – 4.827 and 1.49 – 6.261, respectively, while it was 2.8 and 5.26-fold higher relative to the Ab^+^-non-viremic sera that showed a S/Co range of 0.541–2.06 and 0.5763–3.283, respectively (*P* < 0.0001/each). Results also revealed that 80.04% (*n* = 357) and 89.01% (*n* = 397) of samples tested against denatured (Fig. 5C) and soluble antigens (Fig. 5D), respectively, were concordant with the findings of the viral RNA quantification results, whether the RNA ( +) Ab^+^-viremic sera or the RNA (-) Ab^−^ sera. Meanwhile, 19.95% (*n* = 89) and 10.98% (*n* = 49) of the tested sera were anti-HCV discrepant, *i.e.,* RNA (-) but Ab^+^, which mostly belonged to HCV-recovered patients, either spontaneously or DAA-responders. The distribution of S/Co values in the patient groups is listed in Table 2.

**Table 1:** Demographic and clinical characteristics of the study cohort

*Parameters*		Seronegative Control (n = 63)			Seropositive Patients (n = 383)		*P-value*
		Healthy (n = 50)	Interferent (n = 13)		Non-Viremic (n = 193)	Viremic (n = 190)	
			HBV^+^ (n = 5)	HBV^+^/CRF (n = 8)	Interferents		
					CRF (n = 5, 2.6%)	DM (n = 2, 1.05%)	
					DM (*n* = 5, 2.6%)		
Gender *(n, %)*							
Male		29 (58%)	3 (60%)	6 (75%)	95 (49.22%)	128 (67.37%)	> 0.05
Female		21 (42%)	2 (40%)	2 (25%)	98 (50.78%)	62 (32.63%)	
Age (Yrs)		43.5 ± 8.56	50.8 ± 4.63	59.2 ± 5.5	45.24 ± 6.24	52 ± 9.3	> 0.05
≤ 50, n (%)		21(42%)	2 (40%)	-	83 (43%)	90 (47.37%)	
> *50, n (%)*		29 (58%)	3 (60%)	8 (100%)	110 (57%)	100 (52.63%)	
HCV-Ab		Negative			Positive		
Log_10_ HCV-RNA (IU/mL)		-			-	5.785 ± 0.84	
≤ *8* × *10*^*5*^*, n (%)*						102 (29.91%)	
> *8* × *10*^*5*^*, n (%)*						88 (28.8%)	
Subtype							
4a/m/o *(n, %)*		-			-	152 (80%)/ 10 (5.26%)/ 28 (14.73%)	
ALT (U/L)		25.27 ± 10.18	38 ± 5.08	45.54 ± 7.2	28.34 ± 8.93	41.59 ± 23.12	< 0.0001
AST(U/L)		26.74 ± 7.27	35.88 ± 6.12	26.74 ± 6.3	25.45 ± 7.76	36.12 ± 19.38	< 0.0001
Total BiL (mg/dL)		0.624 ± 0.1	0.824 ± 0.21	1.24 ± 0.32	0.722 ± 0.27	0.764 ± 0.26	0.003
S/Co value	*Denatured antigens*	-			1.22 ± 0.39	2.702 ± 0.79	< 0.0001
	*Soluble antigens*				1.884 ± 0.66	3.753 ± 1.02	

CRF: chronic renal failure, DM: diabetes mellitus, ALT: alanine aminotransferase, AST: aspartate aminotransferase, BiL: bilirubin, S/Co: signal/cut-off

**Table 2:** Distribution and concordance of S/Co values with viral RNA quantification results in patients’ groups

Groups (n = 446)	Distribution of S/Co values among patients’ groups			
	Denatured Ags		Soluble Ags	
	> Predictive S/Co	< Predictive S/Co	> Predictive S/Co	< Predictive S/Co
Number of subjects	182 (40.84%)	264 (59.19%)	275 (61.66%)	171 (38.34%)
Concordant	122 (67.03%)	235 (89.01%)	241 (87.64%)	156 (91.23%)
Discrepant	60 (32.97%)	29 (10.98%)	34 (12.36%)	15 (8.77%)

### Clinical utility of the soluble antigen-based immunoassay

#### Predictive capacity of active HCV viremia

**Fig. 6:**
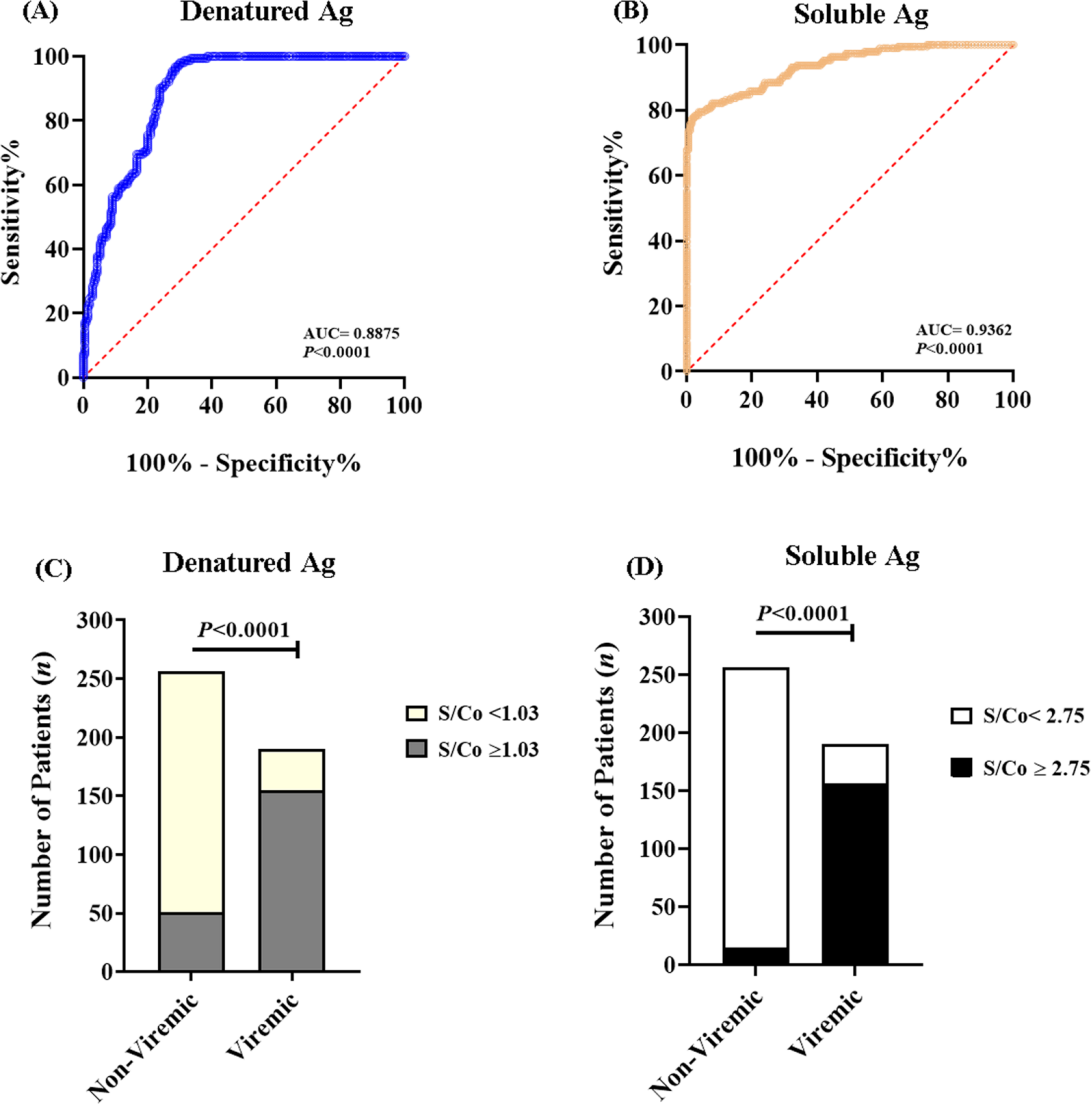
The clinical utility of the denatured and soluble antigens-based immunoassays. **A**, **B** ROC curve analysis of S/Co values of viremic/non-viremic sera assessed against denatured and soluble antigens showed an area under the curve (AUC) of 0.8875 and 0.9362, respectively (*P* < 0.0001/each). **C**, **D**: The best S/Co cutoff values for predicting active viremia was 1.03 and 2.75, respectively, showing 80.08, 94.14% and 81.58, 82.11% PPV and NPV, respectively, with 87.64 and 85.42, 87.64% and 75.24, 91.23% sensitivity and specificity, respectively

To further validate the antigenic utility of recombinant protein preparations, the S/Co values of HCV-Ab^+^ non-viremic patients were used against those of the viremic to calculate a receiver operating characteristic (ROC) curve to assess the possibility of predicting active infection. The analysis showed an area under the curve (AUC) of 0.8875 and 0.9362 for denatured and soluble antigens, respectively (*P* < 0.0001/each). The best cutoff of the S/Co value for predicting active viremia was 1.03 and 2.75, respectively, where higher S/Co values are more likely associated with active HCV viremia. The calculated cutoff values showed 78.81, 82.11% sensitivity, and 78.57, 92.23% specificity, respectively (Fig. 6A, B). These findings were further validated with Fisher’s exact test (Fig. 6C, D), which revealed a higher predictive potential of the S/Co cutoff calculated for serum samples spiked against the soluble coating antigens compared with the denatured, as indicated by higher sensitivity, specificity, positive and negative predictive values, and test accuracy **(**Table 3**)**. Moreover, the soluble antigens showed a marked ability to detect active viremia in sera with viral RNA load as low as 3800 IU/ml in 1/1600 diluted serum sample, while the minimum detection limit of denatured antigens was 19,300 IU/ml in 1/300 diluted sample.

**Table 3:** Antigenic performance of denatured and soluble recombinant HCV-core and E1/E2 proteins

	Denatured antigens	Soluble antigens
**AUC**	0.8875(95% CI: 0.8548 to 0.9202)	0.9362(95% CI: 0.9132 to 0.9593)
ROC curve		
Cut-off value	1.03	2.75
Sensitivity	78.81% (95% CI: 71.62% to 84.57%)	82.11% (95% CI: 76.04% to 86.90%)
Specificity	78.57% (95% CI: 72.53% to 83.58%)	92.23% (95% CI: 87.57% to 95.23%)
Likelihood ratio	3.678	10.56
Fischer’s exact test		
Sensitivity	85.42%(95% CI: 0.8039 to 0.8932)	87.64%(95% CI: 0.8322 to 0.9102)
Specificity	75.24% (95% CI: 0.6892 to 0.8064)	91.23% (95% CI: 0.8603 to 0.9461)
PPV	80.08% (95% CI: 0.7476 to 0.8451)	94.14% (95% CI: 0.9056 to 0.9642)
NPV	81.58% (95% CI: 0.7546 to 0.8645)	82.11% (95% CI: 0.7604 to 0.869)
OR	17.8 (95% CI: 11.07 to 28.65)	73.72 (95% CI: 38.33 to 134.4)
Relative risk	4.347 (95% CI: 3.24 to 5.941)	5.261 (95% CI:3.921 to 7.198)
Likelihood ratio	3.45	9.991
Accuracy	80.72% (95% CI: 76.74% to 84.28%)	89.01% (95% CI: 85.74% to 91.76%)
Lowest detection limit (IU/mL)	19,300	3,800
Concentration of coating Ag/well	250 ng of each Ag	200 ng of each Ag
Lowest reactive serum concentration	1/300	1/1600

95% CI: confidence interval, PPV: positive predictive value, NPV: negative predictive value, OR: odds ratio, IU: international unit, Ag: antigen

#### Correlation between the S/Co values and golden standards of HCV diagnosis

**Fig. 7:**
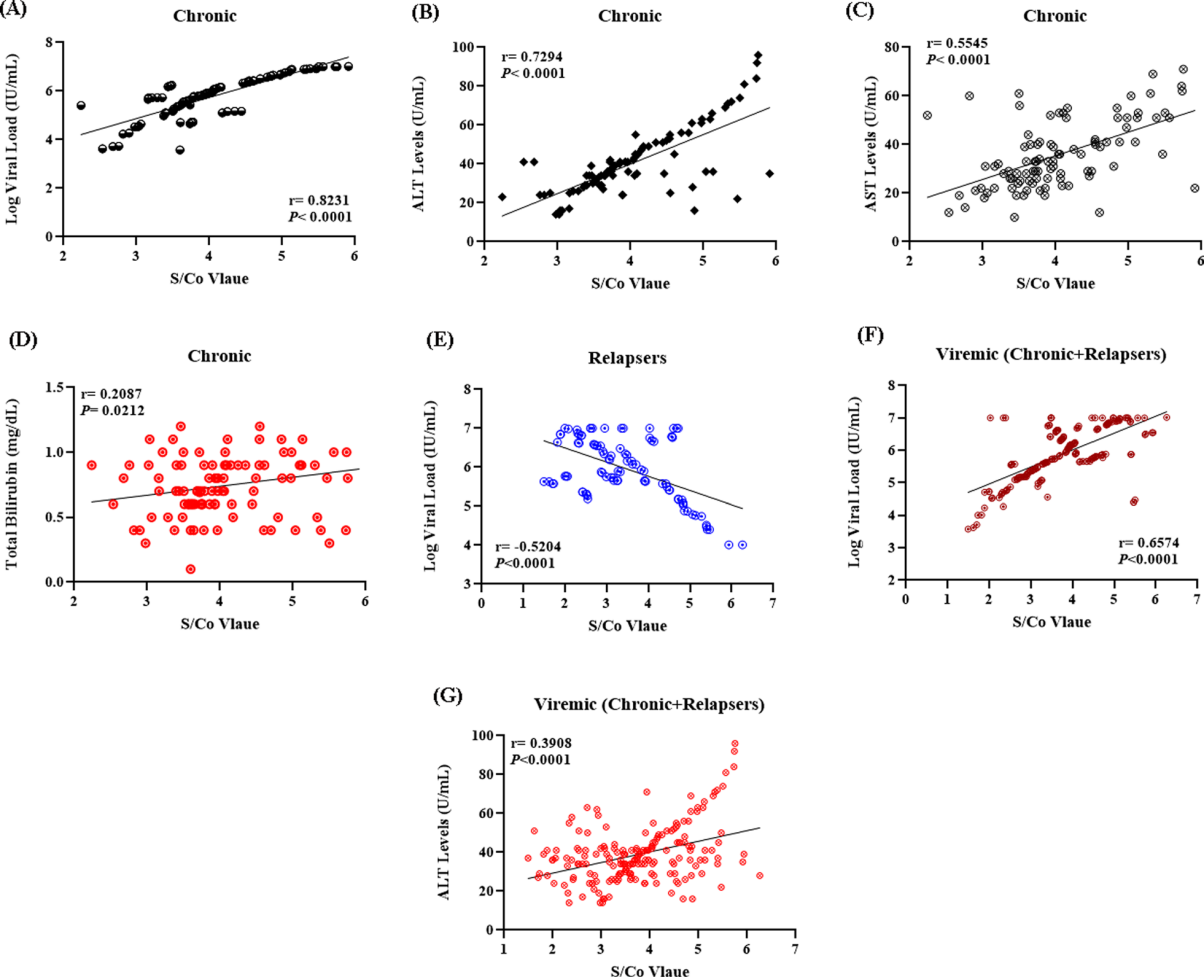
Correlation between the S/Co values and golden standards of HCV diagnosis. No significant correlations were observed between the S/Co values of sera assessed against denatured antigens and other clinical parameters, while those obtained from the soluble antigen-based immunoassay showed positive correlations with viral load, liver transaminases as well as total bilirubin levels in chronic/treatment-naïve patients **A**-**D**, and were inversely correlated with the viral load in relapse /treatment-failure sera (**E**). When tested in the whole viremic group, the S/Co values were positively correlated with both viral load (**F**) and ALT levels (**G**)

As treatment-failure/relapser patients’ sera showed significantly lower S/Co values compared to chronic/treatment-naïve (*P* < 0.0001), the correlation between obtained S/Co values with viral load and/or liver transaminases was first tested in each viremic group individually, and then reassessed in the whole viremic group against the non-viremic. In both assessments, no significant correlations were observed between the S/Co values obtained in the denatured antigens-based immunoassay with any of the other clinical parameters, while the S/Co values of treatment-naïve viremic sera tested against soluble antigens showed strong positive correlations with viral RNA load (r = 0.8231, *P* < 0.0001), ALT (r = 0.7294, *P* < 0.0001), AST (r = 0.5545, *P* < 0.0001), and total bilirubin levels (r = 0.2087, *P* = 0.0212) (Fig. 7A–D). Meanwhile, the S/Co values of treatment-failure/relapsers’ sera showed a relatively stronger inverse correlation with viral RNA load (r = -0.5204, *P* < 0.0001) (Fig. 7E), and positive correlations with both viral load (r = 0.6574, *P* < 0.0001) and ALT levels (r = 0.3908, *P* < 0.0001) when tested in the whole viremic group (Fig. 7F, G).

## Discussion

Although *E. coli* is generally a well-opted platform for the overproduction of recombinant antigens as heterologous proteins, the optimization of vector-host combination and expression conditions to maximize the yield of functional proteins remains empirical. In this study, we aimed to optimize the overproduction of soluble immunoreactive HCV core/envelope proteins in an *E. coli* (BL21)-based system to establish a novel point-of-care immunoassay for active HCV diagnosis with high sensitivity and specificity. To achieve this goal, the full-length core and envelope genes were amplified from local viral strains, and their sequences were verified through BLAST analyses that revealed a partial homology with previously published HCV isolates. Interestingly, the amino acid sequence of the core amplicon showed a maximum homology of 89.06%, which signifies a novel isolate. Considering the high conservation per genotype that renders the HCV core reliable for genotyping/subtyping [25], this finding could be particularly alarming as it implies the emergence of novel viral isolates that might be associated with potential DAA resistance, since this isolate was amplified from treatment-failure patient, although such resistance is usually associated with the polymorphism with drug-targeted genes, *i.e.* NS3 and NS5 [26].

Having discussed the cloning success, it is notable that the number of positive E1/E2 transformants was limited compared to the core. This observation could signify a basal expression of the E1/E2 protein reported with hydrophobicity-associated toxicity [27], despite using a moderate promoter (T5)-based expression vector [28]. Accordingly, several conditions were tailored in glycylglycine-free cultures to overcome this toxicity and obtain the maximum yield of the recombinant E1/E2 protein, including the tight control of basal expression by adding 1% (v/v) glucose [20], low pre/post-induction incubation temperature (30 and 16℃, respectively), and low IPTG concentration (0.2 mM). Yet, the protein profiles visualized on SDS‒PAGE showed several bands of both target proteins at higher molecular weights than expected, suggesting that the rate of expression still favors the protein segregation accumulation in insoluble inclusion bodies. These outcomes might be related to the unregulated protein expression under the power of the consecutive T5-promoter that allows continual transcription of the associated inserts [28]. Such accumulative effect was largely precluded by the integration of glycylglycine in the growth culture that showed concentration-dependent improved solubility of recombinant proteins as indicated by their SDS-PAGE profile, however, in a range-limited manner. While the exact mechanism of glycylglycine in this context is unclear, it could be proposed that high concentrations of the dimer in the growth culture might generate an osmophobic effect, which would induce the overexpression of the heat shock chaperones like DnaK and GroEL(S) in *E. coli* that subsequently promote a ubiquitin–proteasome pathway and/or autophagy-mediated degradation of cytosol-accumulated proteins [29–31]. By the same logic, the preferential promotive effect of glycylglycine observed on the cell density and protein yield in the recombinant E1/E2 cultures, despite the reported protein toxicity [27], could be related to the preferential recognition/binding of DnaK with hydrophobic motifs of five residues [32] that are typically abundant in the E1/E2 transmembrane (TM) domains [33, 34]. This hypothesis also justifies the divergence between our results and those of Rani et al*.* [21] who recently reported the incompetence of glycylglycine to circumvent the aggregation of recombinant human glycerol kinase (GK) protein despite the convergent expression conditions, shedding more light on the potentially determinant role of the protein structure per se in successful protein solubilization.

Building on the earlier discussion of glycylglycine's impact, it's also plausible that it might assume a direct chemical chaperone-like role by interacting with the misfolded protein backbone through its polar surface, which increases the free energy of the misfolded state and prompts the folding equilibrium toward the native state [35]. This hypothesis was consolidated with the reported ability of *E. coli* to transport glycylglycine, among other di-/oligopeptides, in a similar manner to the ATP–dependent shock-sensitive transport systems [36], which consumes considerable energy and accordingly slackens the rate of metabolic processes including protein synthesis, providing enough time for correct protein folding [37]. Therefore, the uncontrolled transport of glycylglycine when present in high concentrations would be detrimental for the cellular growth/metabolism, which explains the absence/limited cell density and protein production observed in 1 M dimer-supplemented E1/E2 cultures.

The effect of glycylglycine on the functionality of soluble proteins was assessed in terms of immunoreactivity with the corresponding antibodies in HCV-seropositive patients’ sera. While both soluble core and E1/E2 proteins were visualized in western blot as homogenous single immunogenic bands at the expected size with minimal background, the insoluble proteins were determined at higher M.wts in line with their SDS-PAGE profile, which indicated the incomplete solubilization of misfolded proteins despite the denaturing conditions of recovery, thusly, the antibodies would merely recognize the surface epitopes of protein dimers/trimers [38–40]. Consistently, all patients’ sera assayed against the soluble proteins in the established immunoassay during the pilot study showed a broader range of OD_450_ values, with a lower cutoff value and higher upper limit compared with the denatured antigens, which consolidates our hypothesis about their limited immunoreactivity.

While the lack of statistically defined endpoint titer determination generally represents a major obstacle in antibody detection-based diagnostics, the usefulness of the signal-to-cutoff ratio (S/Co ratio) for predicting HCV infection has been approached in several studies [41–44]. Yet, most of the reported S/Co values that predict active HCV viremia were discrepant, with high cutoff values range between 5 and 21 [41, 44]. Besides the potential incidences of false positive results associated with such high S/Co threshold [42, 44], it may also produce false negatives especially during the seroconversion period and/or in immunocompromised patients. In the same line, the OD_450_ of patients’ sera assayed in both denatured/soluble proteins-based immunoassays were normalized by calculating the S/Co values to nullify the effect of discrepant cutoffs, and further assess the antigenic performance of both antigen preparations against different panels of sera. Overall, the soluble proteins demonstrated significantly higher antigenic performance as indicated by 33.24% higher concordance and 82.63% lower discrepancy with the viral RNA quantification results, with no impact of the interferent immunocompromised and/or HBV co-infection observed. Moreover, the soluble proteins-based immunoassay demonstrated higher predictive capacity of active HCV infection despite the higher value of predictive S/Co threshold obtained from the ROC curve analysis, where it demonstrated higher sensitivity/specificity, accuracy, positive/negative predictive values, as well as 80.3% lower detection limit of active viremia in minimal concentration of sera required for detection compared with denatured proteins.

Turning to the clinical implications of our findings, the utility of soluble proteins is further underscored by the observed strong positive correlations between the S/Co values of viremic sera tested against soluble antigens with the viral RNA load and liver transaminases, which overcomes the common drawback of most anti-HCV serological assays that do not often provide results that correlate with the golden standards of HCV diagnosis, particularly in seronegative patients with other hepatic conditions [45]. While we have highlighted several strengths of our developed immunoassay, it is important to consider potential limitations, such as its dependency on the seroconversion rate after the infection onset; therefore, it might require a wider serologic window when compared with molecular diagnostics [46]. Nevertheless, the high sensitivity and low detection limit of the developed system reflect its promising diagnostic potential during the early (acute) phase of infection and/or in immunocompromised patients.

## Conclusion

Herein, we developed an optimized platform for the overexpression of soluble HCV structural proteins in *E. coli* BL21 (DE3). The presence of glycylglycine dipeptide in the growth medium promoted the cell biomass as well as the yield of purified soluble proteins in a concentration-dependent manner. The soluble proteins demonstrated higher immunoreactivity and antigenic performance in a newly developed Ab-based immunoassay, showing a significant diagnostic efficacy of active HCV infection with a significant clinical utility, and providing a cost-effective alternative for quantitative PCR to diagnose and/or follow-up viremic patients. The applicability of the proposed platform extends beyond the reported biomedical approach, where the integration of glycylglycine in the growth medium, minimal inducer concentration, and low induction temperature altogether combined with the virtue of weak/constitutive promoter (T5)-based expression vector enables the stable production of soluble/active proteins, even those with reported toxicity. This could be adventitious in several industrial domains, including drug delivery protein-based polymers, antibodies, enzymes, hormones, anticoagulants, as well as biosurfactant-based waste management.

### Limitations of this Study

As a result of limited funds, we couldn't perform a prior confirmation of recombinant core/E1-E2 proteins using anti-His tag antibodies. However, the interesting findings of the established expression platform encourages us to.

include this assay in our forthcoming investigations in this domain.

## Materials and methods

### Amplification of HCV structural genes

Sense and antisense primers required for the amplification of structural genes (core and E1/E2) of HCV genotype 4 were designed according to published sequences in the HCV database (http://hcv.lanl.gov/content/hcv-db/index) **(**Table 4**)**. Viral RNA was extracted from 200μL of viremic patients’ sera using QIAamp Viral RNA Mini Kit (QIAGEN, Hilden, Germany)according to the user manual, and then it was reverse transcribed into cDNA using Maxima Reverse Transcriptase (Thermo Fisher Scientific, USA). Gene amplification was carried out in two rounds to enhance the amplification specificity; the 1st round was carried out using outer primer sets flanking the target regions, followed by a second round using the amplicon of the 1st PCR as a template and specific gene primers. The PCR design was as follows: 1st PCR was carried out using Hotstart Phusion Taq Polymerase (QIAGEN, Hilden, Germany), 5μL of cDNA was used as a template, and the rest of the reaction mix included 10 μL of 10X PCR Buffer, 1μL of dNTPs (1 mM), 200 nM final concentration of each primer, and 1μL of Hotstart Phusion Polymerase (5 U/μL), and the volume was completed to 50 μL with ddH_2_O (nuclease-free). The cycling conditions were set as one pre-denaturation cycle at 98°C for 30 s followed by 35 cycles of denaturing at 98°C for 10 s, annealing at 62°C, extension at 72°C for 1 min, and a final extension cycle at 72°C for 10 min. The second PCR was carried out using the amplified fragment in the 1st PCR with gene-specific primers with the same reaction components and cycling conditions as mentioned above. PCR products were visualized on agarose gels (1%) premixed with 0.5 µg/mL of ethidium bromide along with a 100-3000bp DNA ladder (Thermo Fisher Scientific, USA). The amplified fragments were purified using a GeneJET gel extraction kit (Thermo Fisher Scientific, USA) according to the user manual.

**Table 4:** List of primers used for the amplification of HCV core and E1/E2 genes

Name	Sequence
Core F outer	5'-CCGGGAGGTCTCGTAGA-3'
Core R outer	5'-GCAGTCATTGGTGACATGGTAGATG-3'
Core F inner	5'-AGCACGAATCCTAAACCTCAAAGAAAAACC-3'
Core R inner	5'-GGGACAGTCAGGCACGAAAG-3'
E1F outer	5'-GTTGCTCCTTTTCTATCTTCCTCTTGG -3'
E2R outer	5'- AATGCAGATGAAGAGGATGGCG -3'
E1F inner	5'-GTTAACTATCGCAATGTCTCAGGCA-3'
E2R inner	5'-CGCCTCAACTTGACTTACCATCATAAACA-3'

### Sequence analysis of the amplified fragments

Viral RNAs and PCR products were sequenced using Sanger Dideoxy Sequencing Technology. The nucleotide sequence blast analysis was carried out using CLC Genomics Workbench 9.5 (QIAGEN, Hilden, Germany) with the default settings. FASTA (https://www.ebi.ac.uk/Tools/sss/fasta/nucleotide.html) and ClustalW (http://www.genome.jp/tools/clustalw/) online tools were used for homology searches of multiple sequence alignments.

### Cloning of amplified genes in the bacterial expression vector

The purified PCR products were first cloned and inserted into the pre-linearized CloneJet PCR cloning vector (Thermo Fisher Scientific, USA) according to the user manual, and then the recombinant plasmids was transformed into *E. coli* DH5α by the heat-shock method [47]. After that, 500μL of SOC medium was added, and the transformation mixtures were incubated at 37°C for 1h at 250 rpm for recovery and expression of antibiotic resistance genes encoded in the recombinant plasmids. After incubation, 100μL of each transformation culture was plated onto 2XYT*^1^ agar media supplemented with carbencillin (50μg/mL) according to Sambrook and Russel [47]. Recombinant colonies were screened by colony-PCR using specific/vector primers to verify the presence and proper orientation of the insert. The positive clones were selected for culture and plasmid extraction using a plasmid DNA miniprep kit (ThermoFisher Scientific, USA) according to the user manual. Both the cloned genes and the expression vector (pQE-Trisystem) (Qiagen, Hilden, Germany) were digested using the FastDigest *KpnI* and *NotI* restriction enzymes (Thermo Fisher Scientific, USA) and then purified and ligated using *T4* DNA ligase (Thermo Fisher Scientific, USA). Five microliters of the recombinant plasmids were transformed into competent *E. coli* BL21 (DE3) cells as aforementioned.

### Optimization of expression protocol

To express the recombinant proteins in soluble forms, individual *E. coli* BL21 (DE3) colonies were grown to saturation in 10 mL of 2XYT culture media containing 100 µg/mL carbencillin at 37°C. One milliliter of this starter culture was inoculated into 100 mL of media containing 100 µg/mL carbencillin, with/without 0.1-1M final concentration of glycylglycine to improve the solubility of expressed proteins according to Ghosh et al*.* [23], and then cultured at 30°C with vigorous shaking at 250 rpm until the OD_600_ reached 0.6–0.8. The expression of recombinant protein was induced by adding isopropyl *b*-D-thiogalactoside (IPTG) to a final concentration of 0.1-1M, and the cultures were incubated overnight induction at 16°C, and 200 rpm, cells were collected by centrifugation at 5000 × *g* and 4°C for 30 min. The recombinant proteins were purified from detoxified cell lysates under native and denaturing conditions using the Ni–NTA fast start kit (QIAGEN, Hilden, Germany). According to the user manual, the native protein purification was performed by resuspending the collected cells in 10 mL of native lysis buffer (pH 8) supplemented with the provided lysozyme (1mg/mL final concentration) and 250 U Benzonase® Nuclease, and incubation for 30 min in ice bath. The cell lysates were collected by centrifugation at 13,000*xg*, 4°C for 20 min, and then allowed to bind with Ni–NTA agarose column pre-equilibrated with 2 volumes of the lysis buffer for 1h at RT. The column was then washed with 4 volumes of native washing buffer (pH 8) and the agarose – bound protein was eluted with native 2 mL of native elution buffer (pH 8). For purification under denaturing conditions, similar steps were performed, however, the buffer composition and pH were different, where the denaturing lysis, washing, and elution buffer (pH 8, 6.3, 4.5, respectively) typically include high molarities of denaturing agents (urea or guanidinium HCl). To remove the urea/guanidinium traces, the purified protein fractions were precipitated with 10% TCA (v/v) and incubated on ice bath for 30 min. The precipitated protein was collected by centrifugation at 13,000*xg*, 4°C for 10 min, washed twice with 1ml acetone, centrifuged. The protein pellet was air dried and resuspended in 2 mL of 50 mM Tris–HCl buffer (pH 8). Finally, the purified recombinant proteins were detoxified using Pierce™ high capacity endotoxin removal spin columns (ThermoFisher Scientific, USA) according to the user manual.

### Characterizing the molecular weights and antigenicity of recombinant proteins

Recombinant protein preparations were resolved by SDS‒PAGE using a Bio-Rad Mini-Protein II Dual-Slab apparatus according to Laemmli [48] for detection of the molecular weights of recombinant proteins compared to the expected values based on the amino acid number in the amplified genes. To assess the antigenicity of recombinant proteins by their ability to recognize corresponding antibodies in patients' sera, western blot analysis of protein samples was carried out in mini-transblot electrophoretic transfer cells (Bio-Rad-USA) according to **Towbin et al. **[49].

### Establishment of the immunoassay format for anti-HCV detection in patients’ sera

#### Human Sera

Sera from 50 healthy individuals without any history of liver diseases, 13 HCV-seronegative (HCV-Ab^−^) patients with interfering conditions (HBV^+^ and/or chronic renal failure (CFR), and 383 HCV seropositive (HCV-Ab^+^) patients were collected through the *Viral Hepatitis Clinic* in *Ahmed Maher Teaching Hospital*-*Cairo, Egypt* in the context of the HCV-screening national campaign *100 million seha* during the period 2018–2019. HCV-seropositive patients were categorized into viremic and non-viremic according to viral RNA quantification results. Liver transaminases, including serum albumin, were estimated in all collected specimens using the corresponding kits of *Biodiagnostic* (Cairo-Egypt) according to the user manual.

#### Viral load quantification in patients’ sera

Viral RNA was extracted using a QIAamp Viral RNA Kit (QIAGEN GmbH, Hilden, Germany) according to the user manual. Purified RNA from each sample was used as a template for PCR amplification using a Verso SYBR Green One-Step qRT‒PCR Kit Plus ROX (Thermo Fisher Scientific, USA). The reaction mixture included 2ng RNA template, 70 nM final concentration of 5’-UTR genotype 4 specific primers F: 5’-ttcacgcagaaagcgtct-3’ and R:3’-ggtgcacggtctacgag-5’, 1.25µL of RT enhancer, 12.5µL of 2 × One-Step qPCR SYBR Mix, 0.25µL of Verso enzyme mix, and the volume was completed to 25µL with nuclease-free water. For amplification, the cycling conditions included a single round of cDNA synthesis at 55°C for 15 min followed by a polymerase activation cycle at 95°C for 15 min and 40 cycles of denaturation at 95°C for 15 s, annealing at 55°C for 30 s and extension at 72°C for 30 s. After PCR amplification, a melting curve analysis was performed by one cycle of denaturation at 95°C for 3 s, one cycle starting at 60°C for 30 s, and finally melting for 80 cycles at 60°C for 10 s with a 0.5°C increment/cycle. Fluorescence data were continuously collected during this heating to monitor the dissociation of the strands and the derivative melting curves were obtained with Rotor-Gene Q Series software 2.0.3 (QIAGEN GmbH, Hilden, Germany).

#### Genotyping

HCV-infected sera were subjected to genotyping using a Versant HCV Genotype Assay (Lipa, Bayer, Germany). Briefly, HCV RNA was extracted as aforementioned, followed by cDNA synthesis using biotinylated random primers specific for the 5'UTR of G4-HCV [50]. The generated biotinylated amplicons were hybridized to immobilized oligonucleotide probes specific for the 5' UTRs of different HCV genotypes that are bound to nitrocellulose strips by a poly (T) tail. After hybridization, unhybridized DNA was washed out from the strips that were then treated with alkaline phosphatase-labeled streptavidin (conjugate), which was then bound to the biotinylated hybrid. The chromogenic substrate (BCIP/NBT) that allows the formation of a purple/brown precipitate upon degradation by alkaline phosphatase of the conjugate was used for visualization of the banding pattern on the strip.

#### Optimization and standardization of the novel immunoassay

A standard EIA platform was used according to **Engvall and Perlman **[51]. The initial assay optimization was carried out with 1μg/mL of purified recombinant core/E1-E2 mixed protein (1:1 ratio, 200ng/each) antigen and a 1:100 dilution of serum sample. Ten samples of HCV seronegative/positive, each, were used for assay standardization. For further standardization, the optimal concentration of coating antigen and the serum sample dilution were determined using a checkerboard platform, with 100 μL of recombinant core/E1-E2 protein mixture diluted to 500, 300, 200, and 100 ng/mL carbonate-bicarbonate buffer (pH 9.6) and incubated overnight at 4°C. The plates were washed four times with PBST/0.1% Tween-20 (pH 7.4) and blocked with 300 μL/well of 3% bovine serum albumin in PBST for 1h at 37°C. Plates were washed once and then incubated with serial dilutions of patients’ sera (1:50–1:6400 dilution range) in blocking buffer for 1h at 37°C. Next, the plates were washed five times with PBST, 100 μL of HRP-labeled goat anti-human IgG diluted to 1:10,000 in blocking buffer was added, and the plates were incubated for 1h at 37°C, followed by washing five times with PBST. Color development was carried out using 100 μL/well of TMB (Thermo Scientific, USA) at RT for 5–10 min, and the reaction was stopped by the addition of 100μL of 2N H_2_SO_4_. The OD_450_ values were determined using 630 nm as a reference wavelength. Each sample was assayed in triplicate. To determine the cutoff value of the assay, a pilot study that included 42 samples of viremic patient sera was assayed against 55 samples of HCV-Ab^−^ sera, including 42 healthy samples and 13 interferents (HBV^+^ and/or CRF), and the mean OD_450_ values were calculated. The mean ± standard deviation (SD) (the upper limit) value was selected as the candidate cutoff value in each group. The color intensity was measured, and signal-to-cutoff (S/Co) values were calculated to measure the concentration of specific antibodies. Sera with S/Co values ≥ 1 were considered reactive [52]. Positive and negative patients’ sera were classified according to the determined S/Co values, and the distribution of anti-HCV antibody levels was evaluated within each group. The OD_450_ values were compared with the viral loads of viremic patients.

#### Specificity and sensitivity

The collected sera were independently assayed to assess the specificity and sensitivity of the developed anti-HCV immunoassay. The analytical specificity was assessed by negative concordance between anti-HCV results (S/Co values) and viral loads and the absence of cross-reactivity with other interfering conditions/co-infections, while the sensitivity was evaluated in three different ways: positive concordance between anti-HCV EIA results and viral loads, patients’ sera with low viremia, and serial dilutions of the sera with defined viral loads.

#### Statistical analysis

The sample size was calculated using G-Power software version 3.1.9.7. The study has two independent groups. The a priori calculation indicated a difference in the viremic group of approximately 3.03-fold compared to the healthy control group. A minimum of 13 subjects was required to be assigned to each study group to achieve an effect size (f) of 3.03 and a study power of 95% (1-β error probe). This number was required to reject the null hypothesis that was further evaluated by a continuity-corrected squared Fisher's exact test, with a probability of type I error (α error = 0.05) and power = 95%. All numerical results were analyzed using GraphPad Prism version 9.5.1 software for statistical significance, one-way ANOVA, Pearson correlation and linear regression tests, ROC curve calculation, and Fisher's exact test.

## Data Availability

The data supporting the findings of this study are available from the corresponding author Yasmine S. El-Abd upon reasonable request.

## Abbreviations

Gly-gly: Glycylglycine
OD: Optical density
S/Co: Signal-to-cutoff
PPV: Positive predictive value
NPV: Negative predictive value
AUC: Area under the curve
SDS: Sodium dodecyl sulfate
IPTG: Isopropyl *b*-D-thiogalactoside
CRF: Chronic renal failure
DM: Diabetes mellitus
ALT: Alanine aminotransferase
AST: Aspartate aminotransferase
BiL: Bilirubin

## Footnotes

1 2XYT* composition (/L): 16gm Trypton, 10gm Yeast Extract, and 5gm NaCl ± 7gm agar
