# Correction: Glycylglycine promotes the solubility and antigenic utility of recombinant HCV structural proteins in a point-of-care immunoassay for detection of active viremia

**DOI:** 10.1186/s12934-024-02622-8

**Published:** 2024-12-23

**Authors:** Heba Shawky, Ashraf A. Tabll, Reem M. Elshenawy, Naiera M. Helmy, Rehab I. Moustafa, Yasser K. Elesnawy, Marwa M. Abdelghany, Yasmine S. El-Abd

**Affiliations:** 1 Therapeutic Chemistry Department, Pharmaceutical Industries and Drug Research Institute, National Research Centre, Dokki, Cairo, 12622 Egypt; 2 Microbial Biotechnology Department, Biotechnology Research Institute, National Research Centre, Dokki, Cairo, 12622 Egypt; 3 National Committee for Control of Viral Hepatitis (NCCVH), Ministry of Health and Population, Cairo, Egypt; 4 Ahmed Maher Teaching Hospital, Cairo, Egypt

**Correction: Microbial Cell Factories (2024) 23:25**

10.1186/s12934-024-02297-1

In this article the affiliation details for Author Yasser K. Elesnawy and Marwa M. Abdelghany were swapped as ‘Ahmed Maher Teaching Hospital, Cairo, Egypt’ and ‘National Committee for Control of Viral Hepatitis (NCCVH), Ministry of Health and Population, Cairo, Egypt’.

But should have been ‘National Committee for Control of Viral Hepatitis (NCCVH), Ministry of Health and Population, Cairo, Egypt’ and ‘Ahmed Maher Teaching Hospital, Cairo, Egypt’.

The original article has been corrected.

